# Social Network Analysis in Farm Animals: Sensor-Based Approaches

**DOI:** 10.3390/ani11020434

**Published:** 2021-02-08

**Authors:** Suresh Neethirajan, Bas Kemp

**Affiliations:** Adaptation Physiology Group, Department of Animal Sciences, Wageningen University & Research, 6700 AH Wageningen, The Netherlands; bas.kemp@wur.nl

**Keywords:** social behaviour, livestock, sensing technology, community structure, information transfer

## Abstract

**Simple Summary:**

Social behaviour of farm animals significantly impacts management interventions in the livestock sector and, thereby, animal welfare. Evaluation and monitoring of social networks between farm animals help not only to understand the bonding and agonistic behaviours among individuals but also the interactions between the animals and the animal caretaker. The interrelationship between social and environmental conditions, and the subtle changes in the behaviours of farm animals can be understood and precisely measured only by using sensing technologies. This review aims to highlight the use of sensing technologies in the investigation of social network analysis of farm animals.

**Abstract:**

Natural social systems within animal groups are an essential aspect of agricultural optimization and livestock management strategy. Assessing elements of animal behaviour under domesticated conditions in comparison to natural behaviours found in wild settings has the potential to address issues of animal welfare effectively, such as focusing on reproduction and production success. This review discusses and evaluates to what extent social network analysis (SNA) can be incorporated with sensor-based data collection methods, and what impact the results may have concerning welfare assessment and future farm management processes. The effectiveness and critical features of automated sensor-based technologies deployed in farms include tools for measuring animal social group interactions and the monitoring and recording of farm animal behaviour using SNA. Comparative analyses between the quality of sensor-collected data and traditional observational methods provide an enhanced understanding of the behavioural dynamics of farm animals. The effectiveness of sensor-based approaches in data collection for farm animal behaviour measurement offers unique opportunities for social network research. Sensor-enabled data in livestock SNA addresses the biological aspects of animal behaviour via remote real-time data collection, and the results both directly and indirectly influence welfare assessments, and farm management processes. Finally, we conclude with potential implications of SNA on modern animal farming for improvement of animal welfare.

## 1. Introduction

Animals often live in social groups, and the social interactions in these groups are a key part of their social environment. A change in environment for the animal will lead to change in immunological, physiological, hormonal, metabolic and behavioural changes. The multidimensional factors associated in this interaction of animal with other animals, as well as environmental systems, are quite complex. Animals have been shown to vary in their social contacts, leading to individual spatial and social associations among them. These are often described as social networks [1]. Social networks structures have led to refining theoretical frameworks that allow a better understanding of the evolution of social interactions between groups and individuals [1,2].

Digitalization allows for new and innovative methods of data collection to study/investigate animal movements and animal interactions [3]. Social network analysis (SNA) is a spatial proximity network, and a method of conducting multi-layered analyses of animal social behaviour across disciplines, which offers a framework in which related interactions can be studied and compared. It provides a quantitative methodology to analyze and objectively observe animal social groups and populations [4]. SNA frameworks allow the observer to relate individual or group actions to macro-movements of farm animals. SNA focuses on the structure of ties within a set of social actors (animals) and is linked. A network has nodes, which represent the individuals, while edges represent their relations (undirected, associations or closeness; or directed, interactions). Social relations can be social associations based on distance, or social interactions (physical, real encounter, actor and receiver), and it cannot distinguish between the types of associations/interactions (i.e., agonistic vs. affiliative behaviour, or socio-positive and socio-negative behaviour).

SNA was previously limited to monitoring animal behaviour patterns via the movement and migration of domesticated animals in pastures, or by tracking the spread of disease through physical contact and natural transference [5]. Prior to the development of sensors, SNA data collection was limited to real-time human observation. The results relied heavily on the testimony of farmworkers and were open to varying degrees of subjectivity [5]. This observation regularly overlooked subtle behaviours, leaving only macro-level phenomena documented. Human observation is a time-consuming approach with significant opportunity for error. The recent development of SNA tools now allows researchers to observe livestock and animal husbandry systems that involve subtle social interactions in farm animals kept in barns. With the introduction of non-invasive sensor data collection, researchers have increased access to animal behavioural patterns with greater monitoring detail over longer periods.

New sensor technologies in this review include devices such as pedometers, image-based sensors, accelerometers, Bluetooth platforms, high-resolution GPS sensors and Wi-Fi positioning devices. The combination of these sensor technologies provides useful platforms in collecting data on the animal behaviours, vocalizations, interactions between individuals and groups, as well as migration movement patterns and the identification of subtle nuances in behaviour, all of which are now visible in greater detail. One crucial aspect of social interaction is the subtle differences between synergistic and antagonistic behaviours during SNA of farm animals. Sensors allow for significantly greater accuracy when recording natural social interactions between farm animals. This minimizes the margin for error and provides a non-invasive opportunity to investigate in greater depth.

In this review, we seek to evaluate and discuss research areas and key findings that have utilized sensors as their primary method for data collection for SNA. 

## 2. Design and Structure of the Critical Review Process 

Materials available for the review and critique of sensor-based research are limited due to the complexity and relative novelty of using sensor-based data collection for the social observation of livestock. Selection and inclusion for this review was restricted to articles published in scientifically backed, peer-reviewed journals or other reputable sources of scientific inquiry. The articles selected for review were only in English. They addressed some aspect or background relevant to the current developmental strategy within animal husbandry and farming or refer to ongoing research or study within the field. Primary focus was given to studies that demonstrate a qualitative approach to data evaluation and a discussion of the implications of their results and methods through a robust comparison of conceptual assessments. We used Scopus, Web of Science and Google Scholar tools to collect the literature cited in this review. The keywords used were social network analysis, social networks, farm animal interactions, cow behaviours and interactions, animal relations. Both the Boolean and individual searches were conducted as part of this study. Only papers published after 2015 were included to keep relevance. Out of the 50 papers cited in this study, 5 were published before 2015.

## 3. Reality Mining Technology

In the field of human behavioural studies, reality mining is an emerging technology for assessing behaviour predicted to change the way humans live [6]. Reality mining rests under the umbrella of digital footprint analysis and is defined as the amalgamation of social behaviour analysis, utilizing sensor-based technologies for data collection.

Reality mining models behavioural patterns by analyzing and categorizing sensor-based data [7,8]. Through evolving technology, miniaturization and wearable options for animal tracking, sensor platforms have made detailed sensor-based data collection of the social lives of livestock and animal husbandry possible. Via continuous accumulation of sensor data and monitoring social interactions between animals and groups of animals, miniaturized tracking tools enable animal scientists and bioengineers to recreate the reality mining approach and adapt the method to investigate social behaviour in animal groups with greater accuracy. 

Specially developed animal-borne technology has been applied to animal groups and individuals to track and chart daily activities and interactions automatically. Through state-of-the-art software, scientists break the results down to minutes and even seconds of data, allowing for greater accuracy during analysis and evaluation [8,9]. The results of such studies strengthen predictive frameworks and help researchers gain a deeper and more accurate understanding of social interactions within livestock and farm animal groups. Researchers can identify potential areas for improvement, address strategies concerning social dynamics in other groups, and assist with practical applications of animal husbandry such as space, feeding efficiency, resource control, animal welfare, and other elements associated with raising livestock.

## 4. Social Sensing and SNA in Domesticated Farm Animal Studies

So far, SNA has revealed several animal behaviours that were not previously known or were poorly researched and understood. SNA, combined with sensor monitoring, can provide critical insights into the phenomenon of cow resting periods. It is a well-known fact that dairy cows prefer to rest by lying down for longer periods than usual following a medical treatment, such as hoofing [10]. Identifying factors such as when and for how long cows consider in choosing a spot to lie down could be explored through network analysis and sensor-based data. A thorough investigation, using image-based sensors, GPS positioning, accelerometers, or combinations of sensors, would provide insight into the triggers, drives, and social and environmental factors that motivate cows toward these specific behaviours. Farmers could then address the consequences of these behaviours in terms of animal welfare and management. 

**Figure 1:**
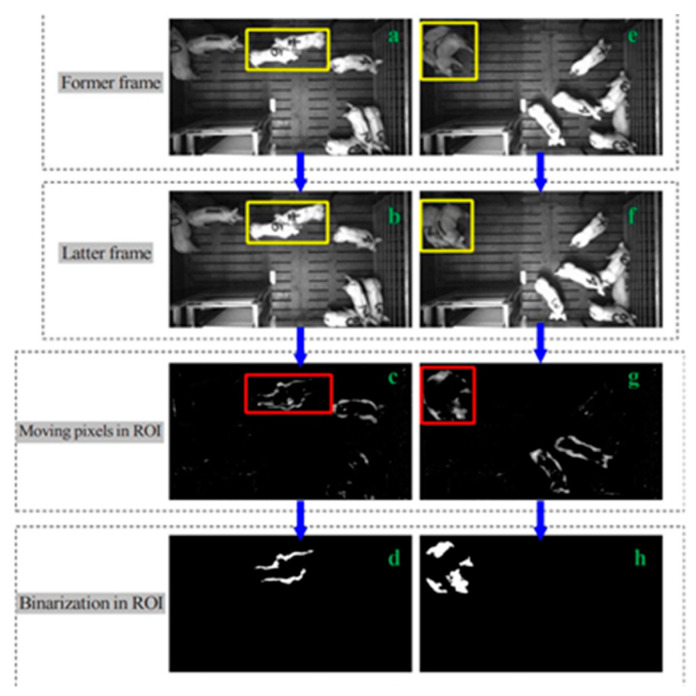
Kinect depth Sensors clarifying behaviours often mistaken as non-aggressive by human observation: (**a**–**d**) aggressive pigs moving slowly, (**e**–**h**) aggressive pigs demonstrating limited activities. (ROI = region of interest). Reprinted with the permission from ref. [11]. Copyright 2019 Elsevier.

What is observed by human observers as one category of behaviour can be easily mistaken for another (Figure 1). To enable researchers and animal handlers to accurately predict social interactions between animals and groups of animals, reliable and consistent data need to be gathered and refined. Sensors play a significant role in facilitating this data collection for SNA. Predictive models of sociological processes, such as the development of hierarchies, information flow, disease transmission, and relationship development could be simplified through an established social framework of competition and cooperation, better known as a binary system. 

It is known that cows use licking for both antagonistic and synergistic behaviours [12]. A mother cow will lick a new-born calf, while a pregnant cow will receive licks from other members of the herd as a sign of support. Meanwhile, cows lick some individuals more than others in an attempt to antagonize and show authority or aggression [12]. This subtle yet powerful behaviour can easily be misinterpreted by the observer, especially if the observation time is shorter. The use of video observation allows licking behaviours to be followed over time and later categorized as either synergistic or antagonistic with greater accuracy. 

The spreading and adoption of new behaviours among animals depends not only on the individual animals that are more and frequently socially connected, but also on other complex functional parameters such as proportion of an individual animal’s social connections to informed and uninformed individual animals [13]. To better understand the socially transmittable behaviours such as anti-predatory, foraging, movements, and sexing in animals, complex contagion concepts should be integrated as part of the SNA. Table 1 demonstrates some fundamental studies using SNA methodology, with several key findings and drawbacks highlighted. 

One of the main challenges faced by dairy farmers is the allocation and availability of calving pens for safe calf delivery. The presence of alien calves affects pregnant dairy cows’ ability or willingness to deliver inside individual calving pens. Social sensor information technology such as image-based sensors or long-term video monitoring could help farmers overcome these practical challenges by detecting micro-cues missed through human observation and assessing activity patterns concerning both the environment and other members of the herd. 

Along with interference in birthing, competition for food in the social groups of cows is a significant concern for dairy farmers. There is a causal link between dairy cows’ tendency to compete for feed and the onset of illness. Foris et al. [14] conducted an investigative study utilizing electronic feed bins that observed how cows balanced the motivation to feed in preferred groups while managing the risk of competitive interaction during feeding. The results showed cows relied on one particular competition strategy, and the consequences reflected the cow’s health on an individual level. Strong, healthy cows maintained their strategies, rarely changing behaviours before or after calving. Whereas cows that developed metritis—a bacterial infection that attacks the uterus after calving—demonstrated a variety of competition strategies upon re-entering the group, most noticeably in the days leading up to diagnosis. Foris et al. [17] evaluated the electronic bins and provide a guidance how to control for technical problems and how to prepare data for SNA in cows. Moreover, they tested in groups to which degree the dominance can be assessed based on electronic bin data from the video using all the cow agonistic interactions. This demonstrates that by monitoring and recording subtle behaviours through sensor data as simple as electronic feed bin monitoring, farmers may be able to detect and treat metritis far earlier than they have previously. 

Tail biting in pigs is a common phenomenon associated with pigs combined in social groups, particularly when concern is not given to their natural dynamics. Li et al. [15] observed tail-biting behaviour in pigs that were separated into two distinct social groups. The pilot study accurately demonstrated a higher incidence of tail biting in pens that contained littermates compared to pens containing non-littermates. The first group consisted of littermates, while the second consisted of non-litter mates. The pigs were recorded via colour-bullet cameras at two time-points 6 weeks and 8 weeks of age for 6 h period, and the footage was reviewed through instantaneous scan sampling every ten minutes. In this study, the researchers counted how often pigs were lying together in each period and created corresponding frequency matrices (lying together (1) or not (0)) which were used for SNA. This potentially indicated that pigs might lose social associations between six and eight weeks of age and that pig communities displayed weak or short-lived social ties overall. Due to the small numbers of pigs observed and significant variations in tail biting, no difference in social network measures was detected [15]. SNA, analyzed with the video and imaging sensor data, has the potential to highlight new phenomena, with more extensive studies needed to be conducted to confirm the evidence. Further studies of this nature may help farming organizations decrease tail biting behaviour within pig communities.

Recent research has highlighted the importance of social behaviours of chickens in animal biology research [18,19]. While they are one of the most important animals for cultivation, they are unfortunately subject to severe welfare issues when contained in agricultural settings. They often display negative and aggressive behaviours, such feather plucking and cannibalism [20]. There is an emerging realization that chickens and other avian species demonstrate social characteristics similar to mammals. Evidence of sophisticated intelligence has been noted in chicken communities [20]. Personality differences in avian animal species correlate with differences in social behaviour [21]. Territorial individual avian species not only influence but also differ in the social network structuring in relation to their personality [21]. Chickens not only have distinct personalities and empathy mechanisms, but they can also exercise self-control [16]. Chickens appear to be highly sensitive to physical touch; demonstrate vulnerability to temperature, pressure, and pain; and also exhibit complex negative and positive emotional cycles in relation to environment, similar to mammal and human physiology [16].

Increased understanding of social structuring could aid egg and poultry producers in helping to reduce incidences of feather plucking and cannibalism, while increasing rates of healthy reproduction to maximize profits. The results also target issues of animal welfare in terms of space allocation and resource management.

## 5. Sensor-Based Data Analysis in Animal Behavioural Studies

SNA provides an opportunity for observational research and analysis of social groups of farm animals and network representations over time. Diverse sensor data collection methods are traditionally employed to monitor different types of social interactions. As a result, depending on which data collection method is employed, various pictures of the social interaction landscape can emerge for both micro- and macro-level analyses.

The ability to identify and categorize conditions that influence animal social behaviour is essential for organizations to implement productive and effective management processes. This is emphasized by information suggesting animal behaviour can be altered by the nature of the social group and the natural environment [4]. The purpose of sensor-based presence systems in farms and animal-based research is to monitor animal behavioural patterns within selected groups and enhance the welfare of animals by catering to personalities and preferences of targeted groups and individuals [13].

Sensor-based data collection has proven effective at recording accurate information regarding animal social behaviour for both the targeted groups and individuals in the group. Sensor-based data collection efficiency is based on the capturing contact frequency and type of the sensor technology used [22]. Recording rate (example: fast moving chickens vs. slow moving cows) and context of animal intercommunications (example: licking vs. tail biting) is a crucial impetus behind refining livestock management processes and maximizing resource distribution and production [5].

In large barns with several animals providing large volumes of data, digitalization becomes essential in the SNA. Gelardi et al. [5] has shown that the sensor-based data collection provides reliability and overcomes subjectivity associated in manual behavioural observations in providing detailed SNA of animal groups. Table 2 outlines some key strengths and weaknesses of introducing sensor-based presence systems to SNA animal behavioural research. Descriptions are provided of the most commonly used forms of “presence” technologies implemented in research studies. The disadvantages and gaps in the research are presented and will later be explored as potential areas for further research and development.

Checking pigs regularly for injury is a fundamental part of the welfare and health assessment of the animals. Failure to detect aggressive behaviour in pigs early in commercial pigsties can limit reproductive capability [26]. Sensor technology has proven to be effective in monitoring micro-behaviours in farm animals that are physical yet non-vocal [26,27]. Lee et al. [23] utilized Kinect Depth Sensors instead of traditional RGB (Red Green Blue) video recordings to monitor aggressive behaviour in pigs, recording instances of physical confrontation such as chasing, knocking, and head-to-head and body contact. The sensors demonstrated a 95.7% detection accuracy and 90% classification accuracy rate for successfully detecting aggressive activity. When the Kinect sensor system based on infrared (IR) technology was used, a real-time depth map was generated with significant advantages over traditional RGB video data currently used by farmers. Accurate RGB footage data deteriorates due to thermal fluctuations during the winter when heat lamps are introduced to the pens; illumination from the lamps interferes with video quality [23]. IR depth sensor technology provided more accurate information in the winter months than traditional RGB stereo-camera methods. 

Proximity sensors are effective tools to monitor the interactions between ewes in herds of sheep [25]. Individual sheep wore the proximity sensors, and proximity generated a maximum of 1 power packet per second when the sensors came close to one another. Power packets indicated proximity, with the lower-power packets used as a proxy for spatial proximity. The sensors enabled researchers [25] to accurately record proximity between sheep that were between 1 to 1.5 m away from each other. The range was set as a detection of close contact between ewes, during which social interactions might occur. Ewes demonstrated a preference towards social ties based on similar characteristics [25], and that this specific preferential choice changed over time. The study revealed no particular community structure among ewes, hierarchical or otherwise. However, aggressive behaviour and social conflict took place when space was reduced and clustering within the community occurred. This became apparent when the ewes clustered to protect themselves in the event of thermal discomfort due to weather changes. The study concluded that the social complexity of ewes is somewhat fluid and influenced by environmental as well as social conditions [25]. The study is one of a few that demonstrated a direct correlation between clustering habits in ewe communities and weather changes courtesy of the sensor data.

## 6. Current Trends in SNA 

Recent research and developments in agriculture have the potential to identify agricultural processes that target specific social aspects of farm animal behaviour, providing foundational methods for assessment and practical implications for research design tools. While there is room for improvement, current studies [26,27] show high precision regarding the monitoring and evaluation of animal social networks. While there are many instances where original sensor-based research has successfully expanded the knowledge base in regard to the social structures of domesticated farm animals, there are still gaps in the literature such as exploration of human–animal relationship that are worthy of consideration for future research.

Sensor-based SNA tools have facilitated the generation of resource management and strategy implementation advices for animal farms. It is important to note, however, that there remains a range of opinions regarding the optimal strategy and approaches for effective agricultural management. Targeted SNA has also been used to conduct in-depth reviews of current applications in preventative animal medicine. Martínez-López et al. [28] conducted a study using SNA methods, which provided data to optimize the current recommendations for farm animal interactions to manage disease-related issues strategically. Real-time monitoring of herd movements would allow for the identification of nodes within a region, allowing for animal disease surveillance and contingency planning, particularly as livestock represents one of the most likely sources for such disease outbreaks.

Freslon et al. [29] assessed the social network process of dairy cows in a manual way without sensor approaches and monitored their relationships regarding disease transmission and control. The report found that as well as tracking the spread of disease, SNA was more successful at observing and recording accurate details about the social hierarchies existing within the dairy cow community than traditional approaches. Not only did the study reveal macro-level social structures within the group, but the method allowed researchers to partially observe subtle attributes and interactions between individual cows. They recorded common traits and gesture types such as sniffing, licking, and rubbing through direct human observation. The outcome helped to optimize the processes of strategically addressing communication trends and behaviours that contributed to the demands of raising livestock, such as surveillance and indicators of social distress. Sensor-technology and the sensor enabled data can improve such analysis and provide insights that are more reliable than the subjective manual scoring and visualization methods.

The act of licking in herds of dairy cows, already considered in Section 4, has been given great emphasis and is considered of principal significance to relationship building and structural management. Machado et al. [12] conducted a study utilizing direct observation and intermittent scan sampling that assessed the preference in partnership selection and sub-group development within dairy cow social structures. They successfully showed that environmental factors played an additional role in animal social welfare, pregnancy and aging and is a fruitful area for continued research and development. As an example of environmental factors that influence the social network of grazing cattle, Salau et al. [10] designed and implemented a study that used video observations of 36 dairy cows in a secluded herd. Cows demonstrated more frequent instances of stress-signalling behaviour when in larger groups compared to smaller groups [30]. The researchers recommended the use of video observations to record variables such as partnership, clique selection and unrest as a potential aid to ongoing research and developments within the agricultural industry. The variables such as partnership and clique selection can also be measured with the aid of new technologies (e.g., GPS or location tracking systems, proximity logger) with proper validation. 

Studies that observe and evaluate the mechanisms of negative behaviours within animal social groups could address gaps in knowledge regarding impacts on resources over extended periods. There is a dire need for integrated software for behavioural monitoring and analysis based on sensor approaches in farm animals for SNA. Investigations into the dominance behaviour and group interactions in pigs highlighted the importance of a nuanced understanding of social interactions [31]. Using video recording, Büttner et al. [32,33] were able to demonstrate the harmonizing effects of aggressive behaviour and physical conflict on the group of pigs. This effect has been systematically overlooked, with agricultural employees traditionally separating animals who display aggressive behaviour due to safety concerns. Similarly, Shizuka and Johnson [34] observed this aspect with the demographic processes influences on the modelling and shaping of social networks. Aggression behaviour of farm animals has been investigated in-depth with the aid of sensor-enabled data. Sensor technologies and sensor data will fill the current gap in SNA in farm animals and will enhance the strategic processes and developmental potentials in animal management and welfare [35,36].

Expert analysts have contributed to the library of recommendations that directly affect farm processes and strategic operations. An extension of sensor-supported SNA studies will benefit the industry, enhancing new resource optimization, the development and innovation of new technologies, and the relationships between individual farm management teams and the evolving landscape of industrial processes and demands on a national level. Farine [37] observed that animal interactions affect social harmony, and the specific findings can be implemented in strategies, such as group creation and separation, network boundary operations, and decision-making processes for animal management, and selection processes. A further study conducted by Puga-Gonzalez et al., [38] determined animal social networks by using null models in combination with a range of statistical methods. The study recommended that GPS technologies, sensor-based location, and tracking data serve as an effective tool to allow farmers to experiment with and emulate ideal environmental conditions, proportional to their resource requirements and profit margin objectives.

Farm-specific research into social networks remains understudied, presenting the most significant gap in the relevant literature, in terms of its research objectives within established boundaries. Combined with social monitoring technology and analysis software, sensor data can be collected in greater amounts, which would lead to an increase in meaningful contributions to enhancing animal welfare [17,23,39]. While the technology is in continuous development and is making breakthroughs in animal husbandry research, analysts acknowledge that the scope of the current sensor-based technological assessment is limited and more research and innovation is needed for continuation and improvement of farm-specific SNA research [31,32,33,40].

## 7. Sensor Data Collection: Challenges and Solutions

Greater understanding and knowledge of the social dynamics of grouped farm animals hold important and complicated implications for both animal welfare and agricultural processes and animal husbandry best practices. Most, if not all, the protocols, methods, and processes of SNA in farm animals were established based on the adaptation of techniques and learnings from SNA of wild animals, but for practical farm management purposes, this adaptation cannot be directly translatable to farm settings. Hence, the precise modelling of SNA in farm animals can only be optimized and validated through sensor-based data. The use of sensor-based investigation has yielded insightful results that can influence how animal handlers, animal caretakers, and agricultural services distribute resources and utilize space. 

One of the main drawbacks of relying solely on sensor information is that proximity sensors cannot detect the context of social interactions. While not an agricultural research project, the Gelardi et al. [5] study is worth considering as an example of the comparative effectiveness of data collection, combining direct observation with automatic sensor data-collection. The study observed one group of primates using sensor-based information collected via both wearable proximity sensors and human observation. The results demonstrated that while some of the directly observed interactions were not detected by the sensors, when the data was synthesized, the results were remarkably similar. The data generated a detailed global map of interactions. Gelardi et al. [5] concluded that sensor data produce reliable results and recommended the incorporation of sensor technology in long-term research initiatives.

Ozella et al. [25] revealed that, while sensors are effective for monitoring clustering behaviours, further indicators of context that influence these behaviours can only be tracked if proximity monitors are used in combination with other sensors, such as accelerometers. While visual analysis of video footage is a valuable tool that allows researchers to observe animals over a greater length of time, there are disadvantages to relying solely on video footage for observation. Salau et al. [10] reported inaccuracies in cow positions on their video analyses as the cows did not move with any kind of uniform speed or direction. They also recorded the footage at lower frames per second (fps); recording for long periods at high fps caused stress on the hardware. Therefore, the team recommended that video observations be conducted only where dense information gathering is required and higher fps are not needed. 

Similarly, in the Lee et al. [23] study discussed previously, the loss of visualization due to lighting conditions made monitoring pig behaviour difficult in winter months. However, the study adequately demonstrated that sensor data collection methods are more than capable of addressing these gaps. Additionally, studies that require a focus on more subtle behaviours between individuals may benefit from a combination of video footage with the advantage of global mapping offered by proximity sensors to provide more accurate results. Video footage can make up for the proximity sensors’ inability to record context in behavioural interactions. The proximity sensors’ ability to accurately record movement, direction, speed, and duration can compensate for the loss of visibility in long-term studies using recordings with low fps. This suggests that sensor fusion and sensor integration is essential for deriving valuable and meaningful insights to understand the context-based indicators in farm animals SNA. Bearing this in mind, there are opportunities for research into the possibility of a new technology that focuses on combining the strengths of sensors and video capture. 

Sensor-based research can assist the evolution of presence systems such as direct observation, camera sensors, heterogeneous sensor fusion, multi-modal sensor systems, and vocal sound audio-sensing platforms. The introduction and eventual evolution of such technologies will allow animal social groups to be observed, uninterrupted by human observers, which ultimately strengthens internal validity and enables researchers to move around and interact within free range and barn environments for the benefit of research requirements. The results of such research could have a beneficial effect on farm processes and agricultural profitability as animal welfare has a direct impact on reproduction success and resource management. 

## 8. Process Optimization within the Livestock Sector

While many studies have assessed animal social dynamics through the application of sensor usage and practice applications, some areas are understudied and require further analysis to benefit livestock treatment and profit improvement for farms and animal husbandry. These areas include potential improvements in profit and animal welfare concerning animal management and other livestock-related software that is utilized by employees within the animal husbandry sector.

Assessment of aspects of social networks in farm animals must take into account fundamental social behaviours within farm animal groups and their effect on farm operations and processes. In designing when and how the SNA tools are to be implemented in farms, the relationship between the individual animal’s behaviour and farm processes, such as (i) allocation and distribution of resources, (ii) the hierarchical structures within animal groups, and (iii) other associated farming practices, must be considered.

Recent SNA studies in the field have revealed a potential for further application and improvements of current sensor-based data collection systems in multiple areas of agricultural practice. Animal interactions and space analysis data have been used to successfully correlate genes and phenotypes with environmental conditions to evaluate social preferences within animal groups [41]. Nutritional strategies have been modelled to monitor social interactions between animals in an attempt to minimize conflict and improve optimized feeding processes [42]. Cow, pig, and chicken behaviours have been critically evaluated, and the variables used to implement improvements in operational processes such as resource management and conflict resolution between hierarchical groups [43]. Research into the nature of animal behaviour can continue with this monitoring, with future studies employed in such a way as to influence and optimize other developments within the industry [44].

Validated agricultural software and sensing platform hardware for SNA have been demonstrated in conducting social networks of livestock with end applications focusing on monitoring resource provisions and implementing processes by considering economic extremities [45]. 

Evaluation of animal social network studies assists the implementation of relevant strategies, rather than relying on theory alone which yields less productivity than the field experimentation results [1,46]. Researchers [46,47,48] have assessed the impact of SNA in animal groups to understand its implications in the agricultural industry’s aims and objectives. SNA not only assists in the implementation of strategic industrial processes and the increase in scientific knowledge but also offers guidance for governmental and private interventions for enabling and introducing effective and innovative technologies in animal husbandry. 

The raising of farm animals, resource allocation, spatial distribution in terms of barn and farm design, and animal husbandry and other associated elements can make use of aspects of sensor-based technology and alter processes that are integrated with different strategies and approaches. This could lead to an increase in the optimization of agricultural farm management directly impacted by animal social behaviour. 

## 9. Future Research Directions

With the emphasis on reducing the dependence on the use of antimicrobials in farm animals, the focus on enhancing biosecurity in livestock production, and the goal to understand the impact of climate change on the ecology of animals, sensor-supported SNA research in farm animals becomes more necessary than ever. Low stocking density in farms is associated with a low disease burden and thereby low antimicrobial usage by the farmers. Much research is needed to explore the sensor technologies’ influence on SNA in precisely managing the optimal stocking density of farm animals. Factors such as outdoor vs. indoor farming systems and its relationship to antimicrobial usage, and the associated animal health variables as part of animal interactions, warrant further research. 

Bonding based on social cognition and communication, associative learning, and habituation decides the development and maintenance of positive animal–human relationships [49]. Animals seeking spatial proximity to animal caretakers is an indicator of rewarding experience, and this behaviour can further be investigated in-depth through SNA tools. Sensor-based automated monitoring and precision farming tools as part of SNA are fundamental in improving human–animal relationships [50].

Sensor-based SNA in farm animals continue to discover new variables and additional knowledge that has a direct impact on the agricultural industry, while adding valuable knowledge that informs and guides the progression and design of innovative methods. The combination of primary and secondary emotion-associated behaviour in individual animals, between animal groups, between species, and even relationships between domesticated farm animals and wildlife are strong contenders for ongoing research into the relational variables between SNA and agricultural processes using sensor data collection methods. The social network effect on the spread of infectious disease has not been fully characterized yet in farm animals. It is warranted that future research will focus on hypotheses concerning the validation of correlation between play behaviour and positive social interactions to robustness throughout the life of farm animals. Social determinants of robust health in farm animals and the characterization of determinants of resilience in creating the robust animals can now be explored through sensor-enabled SNA.

The current shortcomings in the study of animal behaviour in the context of daily agricultural processes are related to the ability to precisely understand animal preferences to minimize resource waste while optimizing the distribution of resources as effectively as possible. This involves keeping animals contented and healthy for as long a period as possible so that farming operations can be streamlined. Researchers currently target the most significant gaps in knowledge for further observation and analysis rather than areas that demonstrate the highest efficiency and effectiveness to emulate processes in other systems. Striving to improve technological systems and research methods to explore and understand animal behaviour in terms of SNA, developers and analysts could strengthen strategic designs that address areas of concern or that are under development within the agricultural industry.

## 10. Conclusions 

Livestock is commonly kept in unnatural group structures, raising questions on animal welfare. Often, welfare is determined by the absence of aggression, yet the well-being of animals is also dependent on the nature of their social interactions and the ability to establish individual bonds. SNA analysis provides elegant tools to assess the social structure of farm animals kept in groups as it also allows to quantify changes over time and consequences of changes in group structures. Utilization of integration of image-based sensors, GPS monitors, proximity sensors, and long-term video monitoring could allow the collection of such sensor fusion data, for better understanding of the behaviour of farm animals. Recent advances in sensing technologies offer the potential to provide critical insights for SNA in farm animals through data-based exploration of the dynamic interactions between individual farm animal’s social connections and the overall adoption of specific behaviours in the farming systems. With the advent of novel wearable sensor modules and biosensor platforms, and data analysis machine learning tools, researchers will continue to explore and make advances in enhancing animal welfare through SNA in farm animals. 

## Figures and Tables

**Table 1:** Social network analysis (SNA) and its implications in farm animals.

Reference	Topic	Unit Analysis	Research Design	Key Findings	Drawbacks
[13]	Spread of behaviours through animal social networks	Literature review	Static and dynamic network analysis	Complex contagions and socially transmitted behaviours can be adequately studied and quantified using SNA	Behaviour spreads through natural populations in intricate patterns, not simply as previously thought
[14]	Competition strategies of metritic and healthy cows	Electronic feed bins and direct observation	SNA related to social competition strategies	Cows with metritis altered competition strategy, while healthy cows registered no change	Due to frequent changes in group composition, information regarding hierarchical establishment was limited
[15]	Understanding of tail biting in pigs using SNA	Infrared colour-bullet camera capture and instantaneous scan sampling	SNA using binary matrices	Pigs displayed weak social connections with both litter and non-littermates	Due to the young age of the animals, social bonds could not be adequately studied
[16]	Review of cognition, emotion, and behaviour in domestic chickens	Literature review	In-depth analysis of referenced empirical and review papers	Evidence that chickens have complex social structures and are behaviourally sophisticated. Distinct personalities detected and evidence of empathy mechanisms	No information on higher-level explanations for social mechanisms, or if social complexities are evident across species

**Table 2:** Strengths and weaknesses of sensor-based data collection in social network analysis in animal behavioural research.

Reference	Topic	Sensor Technology	Key Findings	Drawbacks
[5]	Measuring social networks in primates	Wearable proximity sensors and direct observation	Sensor data yielded results similar to direct observations	Sensors did not detect all observed interactions
[10]	Dairy cow contact networks	Automated video footage of 36 penned dairy cows	Structure of cow cliques changes before and after milking	Only visually detected cow movements were recorded
[12]	Lick and agonistic interactions between dairy cows	Direct visual observation combined with intermittent scan sampling	Cows engaged in agonistic interactions more often with preferential herd mates—links between licking and recent activity. Pregnant cows showed an increase in received licks	Relevant data not captured to analyse cows who “requested” licks
[23,24]	Automatic recognition of aggressive behaviour in pigs	Top-view Kinect camera and Kinect depth sensors installed over the pigsty	The method was effective for accuracy and cost-effectiveness	Restrictions when monitoring the behaviour of single pigs
[25]	The effects of age, environment, and management on social contact patterns of ewes	Wearable proximity sensors in correlation with climatic indices	Positive correlation between social clustering, characteristic similarities, and environment. Potential to enhance management systems in a production setting	Proximity sensors did not record behavioural context
